# See your GP, see the world – An activating course concept for fostering students' competence in performing vaccine and travel consultations

**DOI:** 10.3205/zma000970

**Published:** 2015-08-17

**Authors:** Esther Beltermann, Sibylla Krane, Jan Kiesewetter, Martin R. Fischer, Jörg Schelling

**Affiliations:** 1 Klinikum der Universität München, Institut für Didaktik und Ausbildungsforschung in der Medizin, München, Deutschland; 2 Klinikum der Universität München, Lehr- und Simulationsklinik, Zentrum für Unterricht und Studium, München, Deutschland; 3 Klinikum der Universität München, Institut für Allgemeinmedizin, München, Deutschland

**Keywords:** communication, counseling, simulation, vaccine and travel consultation

## Abstract

**Aim: **Performing vaccine and travel consultations is a crucial aspect of the daily routine in general medicine. However, medical education does not provide adequately and structured training for this future task of medical students. While existing courses mainly focus on theoretical aspects, we developed a course aiming to foster practical experience in performing vaccine and travel consultations.

**Project report:** The course was implemented in the simulation clinic at the University of Munich in the summer 2011 semester using role-plays in a simulation-based learning environment. The course represents different disciplines involved in vaccine and travel medicine. Students’ learning is supported through active engagement in planning and conducting consultations of patients.

**Discussion and Summary: **The course was implemented successfully and students’ acceptance was high. However, there is a need for structured teaching of theoretical basics in vaccine and travel medicine earlier in medical curriculum. The insights gained through our course are used for the development of the structured longitudinal curriculum “vaccine medicine”.

## Zusammenfassung

**Ziel:** Impf- und Reiseberatungen sind eine wichtige Aufgabe in der allgemeinmedizinischen Praxis. Bisher wurden Medizinstudierende darauf nur wenig und uneinheitlich vorbereitet. Während bestehende Formate meist theoretische Grundlagen verfolgen, fokussiert dieses Wahlpflichtseminar die Förderung der praktischen Durchführung von impf- und reisemedizinischen Beratungsgesprächen.

**Projektbeschreibung:** Im Sommersemester 2011 wurde dieses neuartige Wahlpflichtseminar in der Lehr- und Simulationsklinik der Universität München eingeführt. Die Seminareinheiten werden fachübergreifend und simulationsbasiert durchgeführt. Die Aktivierung der Studierenden und Förderung des Lernerfolgs wurde durch ihre eigenverantwortliche Planung und Durchführung von Beratungsgesprächen mittels Rollengesprächen erreicht.

**Diskussion und Schlussfolgerung: **Das Seminar wurde erfolgreich eingeführt und die Evaluationsergebnisse sprechen für eine langfristige Implementation dieses praktischen Kurses. Allerdings sollte ein strukturiertes Grundlagenwissen zur Impf- und Reisemedizin bereits systematisch im medizinischen Curriculum gelehrt werden. Die Erkenntnisse fließen in ein neu entwickeltes longitudinales Curriculum „Impfmedizin“ ein.

## 1. Introduction

Vaccinations serve an important and effective preventive measure to decrease the incidence of diseases [1]. Achieving broad immunization coverage contributes to individual protection, protection of susceptible groups of patients and elimination of germs [2]. Thus, physicians are requested to ensure a satisfactory level of immunization which is comprised of the primary immunization, and the preservation of immunization through repeated vaccinations, vaccinations according to indications and travel vaccinations [1]. Physicians should be aware of the benefits and typical risks and complications of vaccinations. Additionally, they should know relevant sources of information that build the foundation for elaborated consultations of patients. Moreover, physicians should possess appropriate communication skills to conduct such consultations [2]. Being prepared for challenging consultations is especially important when considering today’s vaccine fatigue and the anti-vaccination movement. 

Despite the importance of vaccine and travel vaccine, medical education at the University of Munich does not provide adequately structured training for this affordance. Existing courses mainly focus on theoretical aspects but neglect the role of practical training opportunities. 

We, therefore, developed a course aiming to foster practical experience in performing vaccine and travel consultations. The course follows clear learning objectives and facilitates students’ active engagement [3].

This article provides an overview on the development and implementation of this course.

## 2. Project report

### 2.1. Goal and didactic concept

In the summer 2011 semester, we offered a compulsory elective subject focusing on vaccine and travel consultations in general medicine for medical students in their third to sixth clinical semester. The overall goal of this course is to enable students to conduct vaccine and travel consultations. This encompasses

1. theoretical knowledge about standard and indicated vaccinations and an overview of the broad field of travel and tropical medicine, as well as
2. information on sources of information in vaccine and travel medicine, and
3. knowledge about consultations and opportunities for practical training.

To achieve these goals, we developed a course that connects theoretical knowledge with practice. Short lectures, lecture notes and textbooks served to impart theoretical knowledge. Acquired knowledge was directly applied in a practical and relevant social context [4], [5], [6]. In line with situated learning [6], the course was conducted in an authentic learning environment representing a doctor’s office in the simulation clinic of our university hospital. Vaccination reports, brochures and a model of an arm to practice vaccinations (Model P55, 3B Scientific) were used [5]. The learning environment corresponds to medical students’ future workplace and its affordances in all its complexity. Following Cognitive Apprenticeship [7], the intramuscular injection is first demonstrated by the instructor and then performed by students increasingly self-independently. Vaccine and travel consultations are simulated in role-plays using authentic cases in which each student takes over the role of the physician or the patient. Each simulation is followed by a structured feedback process that provides students with a realistic impression of their competence and allows for the integration of experiences in their action repertoire [6], [8]. 

#### 2.2. Implementation

**Figure 1:**
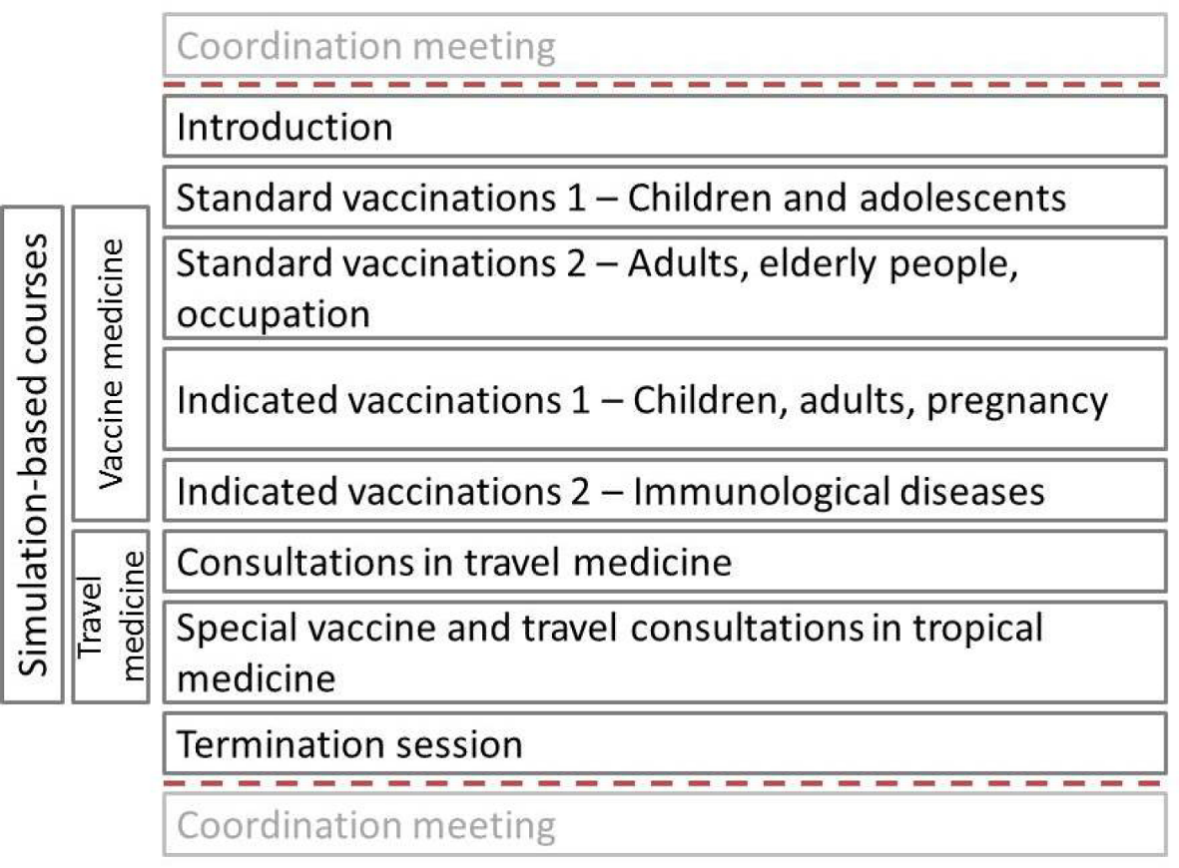
Curriculum

The course was offered as part of the compulsory elective curriculum and consists of eight sessions, each lasting 120 minutes (see Figure 1 (Fig. 1)). The number of participants is limited to ten students. 

The first session serves as an introduction and overview of the learning objectives, course schedule and organizational aspects and addresses some theoretical basics. Moreover, the prototypical course of a vaccine and travel consultation is introduced and is comprised of a greeting, taking patient history with an emphasis on previous vaccinations and documentation of vaccinations, identification of the need for prophylaxis, providing information on vaccination, planning prophylaxis, documentation, and discharge [9].

**Figure 2:**
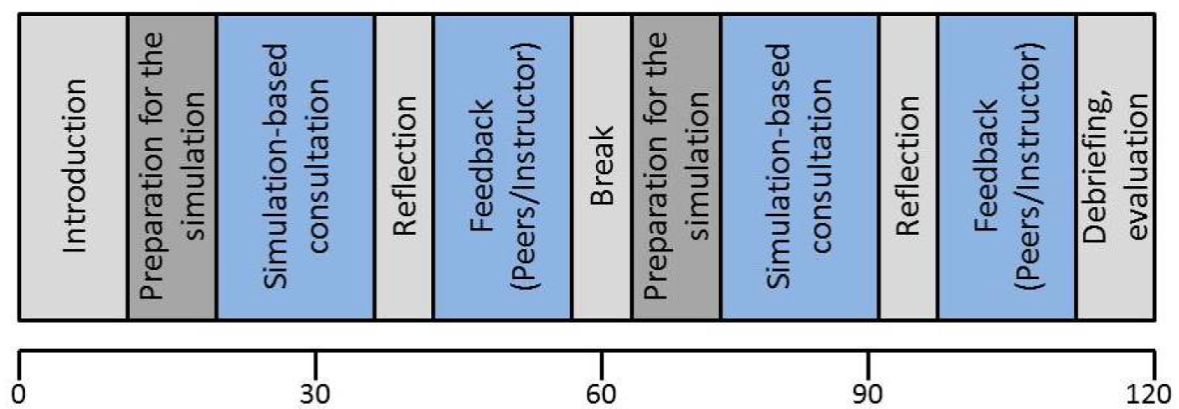
Process of the sessions

The following six sessions focus on practical training and are organized around the core areas “vaccine medicine” and “travel medicine”. The sessions on vaccine medicine focus on standard and indicated vaccinations while the sessions on travel medicine address general and specific consultations related to travelling. All sessions follow a standardized process (see Figure 2 (Fig. 2)).

Each session is conducted by one of our six instructors who have extensive professional and teaching experience in their core area of vaccine and travel medicine (e.g., general medicine, tropical medicine). Each instructor spends 120 minutes per semester on this course. A coordination meeting with all instructors at the beginning and the end of the semester serves organizational aspects. 

## 3. Evaluation of the course

Since the first implementation in the summer semester 2011, 31 students participated in this course (N_SS11_=4; N_WS11_=8, N_SS12_=10, N_SS13_=9). The course was evaluated at the end of each session with a return rate of 80 percent. The evaluation sheets based on a modified version of the SFDP26-German [10] consists of three areas:

1. perceived learning success,
2. execution of the simulations and
3. assessment of the course.

A text box allows for individual comments. Students’ satisfaction with the course has been very high and students have reported a growth in theoretical knowledge and practical skills (see Table 1 (Tab. 1)). Both the course concept and the instructors were assessed very positively (“Great course on vaccine and travel medicine”, “Great instructors”). However, students’ comments also indicated a need for a better theoretical knowledge base (see Table 1 (Tab. 1)). 

## 4. Discussion

Our experiences with this course are positive. The development of this course and the extension of the team of instructors since the first implementation in the summer 2011 semester positively contributed to the quality of this course. The interdisciplinary team of instructors accounts for the heterogeneity of vaccine and travel medicine. Students could benefit from their comprehensive experience in their core area in vaccine and travel medicine. However, the extension of the team of instructors requires a clearer structure and connection of contents.

The results of the evaluations confirmed the relevance of this course. They indicated both medical students’ learning success and high interest in vaccine and travel medicine. However, students’ wish for a better theoretical knowledge base also pointed to the necessity of a more systematic medical education in this area which our course alone cannot ensure. Building on such a sound theoretical knowledge base, this course, nevertheless, provides an excellent opportunity to apply theoretical knowledge in a practical and authentic context and to learn how to conduct structured consultations in vaccine and travel medicine. Our insights are used for the development and implementation of an interdisciplinary longitudinal curriculum “vaccine medicine” at the medical faculty of the University of Munich.

## Competing interests

The authors declare that they have no competing interests.

## Figures and Tables

**Table 1:**
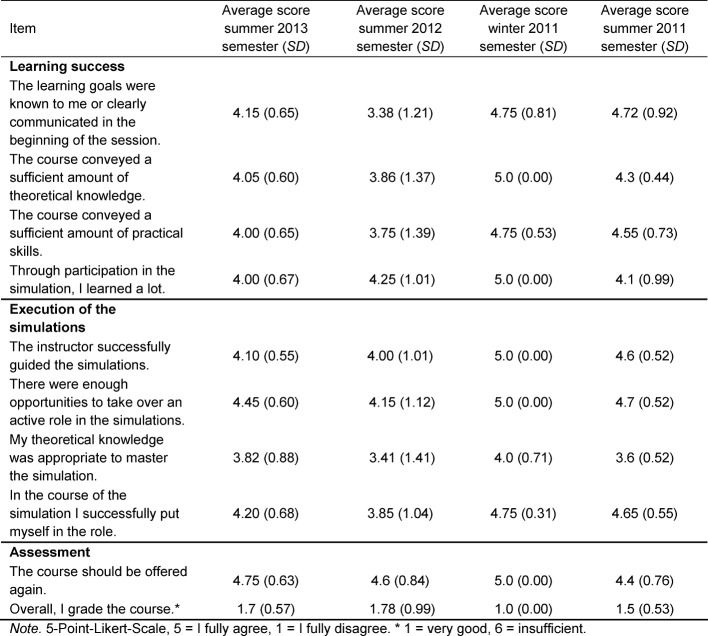
Evaluation
